# The Efficacy of Ultrasound-Guided Paravertebral Block in Laparoscopic Cholecystectomy

**DOI:** 10.3390/medicina54050075

**Published:** 2018-10-23

**Authors:** Gülçin Aydin, Oktay Aydin

**Affiliations:** 1 Department of Anesthesiology and Reanimation, Kırıkkale University School of Medicine, 71450-Kirikkale, Turkey; 2 Department of General Surgery, Kırıkkale University School of Medicine, 71450-Kirikkale, Turkey; droktayaydin@gmail.com

**Keywords:** ultrasonography, ultrasound, peripheral nerve block, ultrasound-guided injection

## Abstract

*Background and objectives:* Despite its wide use in thoracic procedures, to date, few studies have assessed the effectiveness of paravertebral block (PVB) in laparoscopic cholecystectomy (LC) in an adult population. In these studies, PVB was performed bilaterally using nerve stimulator guidance. To the best of our knowledge, the effectiveness of unilateral preoperative and postoperative ultrasound-guided PVB has not been evaluated in patients undergoing elective LC. The aim of this study was to evaluate the efficacy of single-dose unilateral paravertebral block (PVB) in patients undergoing laparoscopic cholecystectomy (LC) under general anesthesia. *Materials and Methods:* Patients undergoing LC were randomly separated into control, preoperative block, and postoperative block groups. PVB was performed unilaterally using bupivacaine under ultrasound guidance. Postoperative pain within the first 24 h, side effects, intraoperative opioid and postoperative analgesic requirements were noted. Evaluation was made of a total of 90 patients (25 males, 65 females) with a mean age of 45.78 ± 14.0 years (range, 19–74 years). *Results:* Opioid and additional analgesic needs and nausea/vomiting rates were significantly reduced in the preoperative block group compared to the other groups (*p* < 0.05). Visual Analog Scale (VAS) scores were significantly lower in the preoperative and postoperative block groups compared to the control group (*p* < 0.05 for all). When the VAS scores were compared between the preoperative and postoperative block groups, a significant difference in favor of the preoperative group was observed in terms of the zero minute-, 1st and 2nd h assessments (*p* < 0.05 for all). *Conclusions:* Ultrasound-guided PVB is a useful and safe approach for pain management during and after LC. Preoperative block can also reduce the rate of requirement for intraoperative opioid and postoperative analgesia.

## 1. Introduction

The mechanism of pain after laparoscopic procedures is thought to be multifactorial. The main causes of pain after laparoscopic cholecystectomy (LC) include pain arising from the incision site, pneumoperitoneum and cholecystectomy [1]. To date, clinicians have investigated various methods for reducing pain after LC. These include non-steroidal anti-inflammatory drugs, intraperitoneal local anesthetics, local anesthetics applied to the wound site, removal of the insufflation gas, paravertebral block (PVB), and epidural block [1].

Paravertebral block, which was first described in the early 20th century, is the administration of local anesthetic into the wedge-shaped space on the antero-lateral thoracic spine in order to provide abdominal analgesia [2]. This space includes spinal and sympathetic nerves and their block provides effective anesthesia in the management of thoracic and/or abdominal pain after various types of surgery [3]. The application of PVB has been shown to improve pain scores and decrease analgesic drug requirements, postoperative nausea, and vomiting [4,5].

Paravertebral block can be performed using ultrasound-guided, stimulation-guided or landmark-based approaches, and can be applied before or after surgery. To obtain an effective analgesic effect, some authors perform multiple injections [2,6,7].

Despite its wide use in thoracic procedures, to date, few studies have assessed the effectiveness of PVB in LC in an adult population [7,8]. In these studies, PVB was performed bilaterally using nerve stimulator guidance. To the best of our knowledge, the effectiveness of unilateral preoperative and postoperative ultrasound-guided PVB has not been evaluated in patients undergoing elective LC. We hypothesized that unilateral ultrasound-guided preoperative PVB would improve the analgesic effect during and after surgery with low side effects/complications. Therefore, this prospective, randomized and controlled study aimed to investigate the effectiveness of ultrasound-guided unilateral PVB with a single dose of bupivacaine combined with general anesthesia and to find the optimal timepoint for the application of PVB in order to obtain the maximum analgesic effect in LC. It also aimed to investigate the additional effect of PV combined with general anesthesia on intraoperative opioid need, postoperative pain, nausea, and vomiting.

## 2. Materials and Methods

### 2.1. Study Design and Participants

The study included 90 patients who underwent LC between January 2018 and June 2018 at Kırıkkale University Medical Faculty Hospital. The patients included were aged 18–75 years, with an American Society of Anesthesiologists Patient Classification (ASA) score of ASA I–III. Using the sealed envelope method, patients were randomly and equally assigned to three groups. A record was made of their demographic characteristics, including age, gender, height, and weight, and ASA category, operation duration, and perioperative opioid requirement. The study was approved by the Local Ethics Committee of the hospital (decision no.: 2018-15/02). Informed consent was obtained from all participants.

Patients who were planned for elective cholecystectomy due to cholelithiasis were included. Patients with acute cholecystitis, acute cholangitis, jaundice, and biliary colic symptoms were excluded. Patients with local infections at the PVB site, hypersensitivity to the drugs used for the PVB, coagulopathy, spinal/paravertebral deformities, morbid obesity, and severe aortic stenosis were excluded from the study [2,6,7]. The minimum sample size was calculated as 67. During the study period, 130 patients underwent LC in our institute and 90 eligible patients accepted to participate to the study.

The standard general anesthesia protocol required for LC was applied to all patients included in the study. No additional procedure except general anesthesia was applied to the patients in Group 1 (*n* = 30). PVB was applied preoperatively to patients in Group 2 (*n* = 30) under sedation with 0.03–0.05 mg/kg midazolam on admission to the operating room, just before the operation, and this was followed by general anesthesia. In Group 3 (*n* = 30), PVB was applied postoperatively to the patients while still in the operating room and under general anesthesia immediately after surgery. The anesthesia was terminated after PVB in this group.

### 2.2. The General Anesthesia Protocol

All patients in all three groups were treated with a standard general anesthesia protocol. The induction of general anesthesia was made with 2–3 mg/kg propofol, 1 mcg/kg fentanyl and 0.5 mg/kg rocuronium. The maintenance of the anesthesia was provided with 2% sevoflurane, 50% oxygen, and 50% air.

### 2.3. The Paravertebral Block under Ultrasound Guidance

**Figure 1:**
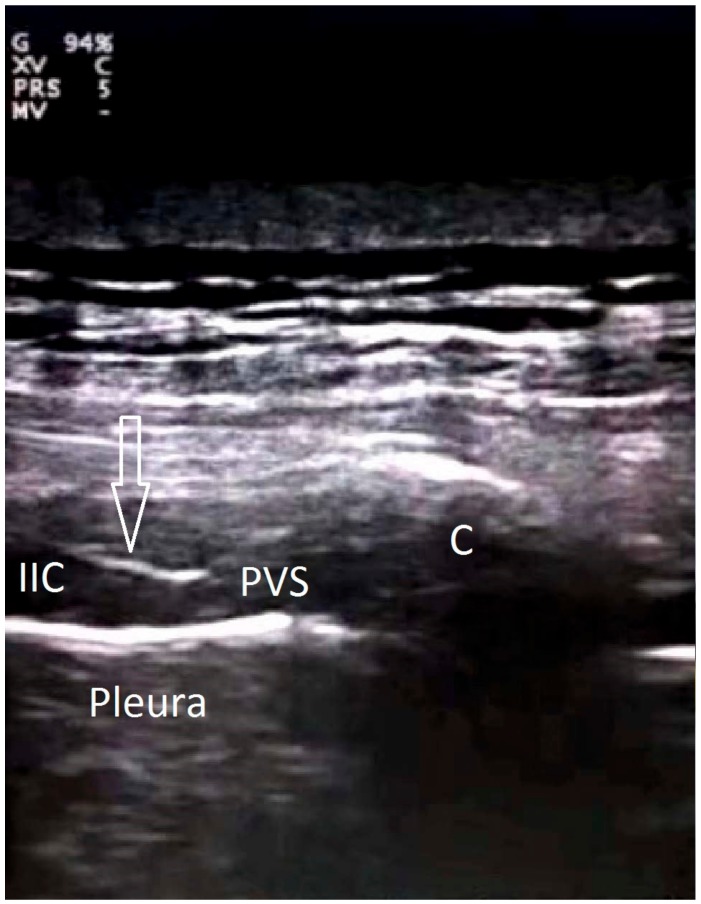
Ultrasound image showing an example injection. EIC: External intercostal muscles; IIC: internal intercostal muscles; PVS: paravertebral space; c: costa; arrows: needle.

The anesthesiologist who had six years’ experience performed the PVB to the patient lied on his one side. A linear 10–18 MHz US probe (Esaote MyLab 30, Geneva, Italy) was placed between two transverse processes sagittally on the paramedian surface, then the transverse processes, superior costotransverse ligament (SCL) and pleura were identified at the level of the thoracic 7 vertebra. The skin and subcutaneous tissue were infiltrated with 2% lidocaine. An 18-gauge 50 mm needle (Pajunk, Geisingen, Germany) was advanced under the US probe using the in-plane technique. After passing through the SCL and reaching the paravertebral space, an aspiration was performed to check for air and/or blood. After negative aspiration, 20 mL of 0.5% bupivacaine was injected. The pleural collapse due to the injected local anesthetic volume was observed. An example image is shown in Figure 1.

### 2.4. Assessments

The required opioid dose during the operation was noted and set according to the depth of the general anesthesia and increased sympathetic activity, i.e., hypertension, and/or tachycardia. The operation duration was accepted as the period between the beginning of the operation and the last skin suture.

The visual analog scale (VAS) for pain, with 0 being the least and 10 being the worst pain during rest, was recorded at 0–1–2–6–12 and 24 h postoperatively.

Possible complications such as hypotension due to sympathetic blockade, nausea and vomiting, and urinary retention during the first 24 h postoperatively were noted. Patients were also evaluated for local complications such as nerve root injury, infection, hematoma, vascular puncture, and pleural puncture. The subjects were treated with intra-muscular (im) 50 mg of dexketoprofen trometamol in cases where the VAS score was ≥4, and if the pain persisted, an additional 50 mg meperidine was administered intra-muscularly. All additional analgesic requirements were noted.

### 2.5. Statistical Analysis

The SPSS (Statistical package for social sciences, IBM, USA) version 20.0 was used for the statistical analysis of the data. Data were stated as mean ± standard deviation (SD), median (25–75%) values or number (*n*) and percentage (%). Numerical variables were compared using One-Way Anova or Kruskal Wallis tests after checking the normal distribution. Post hoc analyses were performed to determine from which group the difference originated. Categorical variables were compared using the Chi Square test or Fisher’s Exact test. A value of *p* < 0.05 was accepted as statistically significant.

## 3. Results

A total of 90 patients (25 males, 65 females) with a mean age of 45.78 ± 14.0 years (range, 19–74 years) were included in this study. The clinical and demographic features of the groups are summarized in Table 1. There were no significant differences between the groups in terms of age, gender, height, weight, ASA scores, and operation duration (*p* > 0.05 for all). Intraoperative opioid requirement, extra analgesic requirement, and nausea/vomiting rates were significantly lower in the preoperative anesthesia group compared to the other groups. Extra analgesic requirement and nausea/vomiting rates were significantly lower in both the preoperative and the postoperative block groups compared to the control group. 

**Figure 2:**
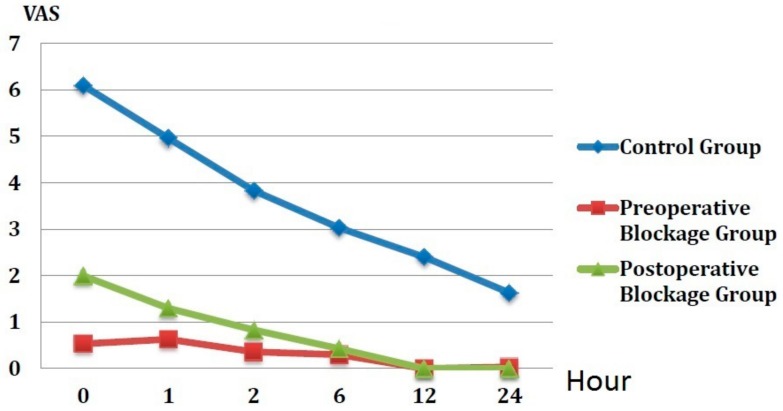
The VAS scores of the groups.

Comparisons of the VAS scores of the groups just after the operation (0), then at the 1st, 2nd, 6th, 12th, and 24th h are shown in Figure 2. There was a significant difference between the groups at each evaluation time (*p* < 0.05 for all). The VAS scores for each evaluation were significantly lower in the preoperative and postoperative block groups compared to the control group (*p* < 0.05 for all). When the VAS scores were compared between the preoperative and postoperative block groups, there was a significant difference in terms of the 0, 1 and 2-h assessments (*p* < 0.05 for all). No significant difference was observed between the 6, 12, and 24-h assessments.

## 4. Discussion

The aim of this study was to investigate the effectiveness of the ultrasound-guided one-point PVB at the thoracic 7 vertebrae level in reducing postoperative pain in patients undergoing LC and to compare the outcomes of the preoperative and postoperative timing of this procedure on pain and intraoperative opioid requirement. The main findings can be summarized as follows: the patients treated with preoperative PVB required no opioids during surgery, and the analgesic effect of preoperative PVB was superior to that of postoperative PVB in the first two hours after surgery. Patients in both the preoperative and the postoperative groups requested less additional analgesia and reported less nausea after surgery. There have been previous reports of the additional analgesic effects of PVB when combined with general anesthesia. Furthermore, the preoperative and postoperative applications of PVB in LC were also compared. To the best of our knowledge, there has been no previous study which has compared ultrasound-guided unilateral preoperative and postoperative PVB and a control group in patients undergoing LC.

Paravertebral block allows for the blockage of ipsilateral, segmental, somatic, and sympathetic nerves. It can be performed using single- or multiple-level injection techniques, unilaterally or bilaterally. To date, clinicians have used different guidance for PVB injections. Initially, PVB injections were performed using a landmark-based conventional technique, but in recent years, the nerve stimulation technique and ultrasound-guided PVB techniques have been performed successfully for pain management after various thoracic and abdominal surgeries [2,6,7,8,9]. In emergency conditions, ultrasound is an imaging modality with advantages such as ease of access, low cost, the possibility of conducting bedside examinations, and the provision of real-time imaging. Ultrasound is increasingly used for guidance in various interventional procedures. Ultrasound-guided nerve blocks have been performed with improved efficacy and decreased complications via real-time visualization of the targeted anatomic structure or space, surrounding structures, and the approaching needle [2]. Shibata and Nishiwaki first described an ultrasound-guided thoracic PVB using a transversal, in-plane approach. Recently, different ultrasound-guided PVB techniques have been introduced [10,11,12,13,14,15]. Ultrasound-guided PVB can be performed with the probe placed transversely or sagittally on the paravertebral area, using in-plane or out-plane techniques. The injections can be performed either lateral to medial in the transverse direction. The ribs, spinous, and transverse processes of the vertebra, costotransverse joints, and pleura are used as sonographic landmarks. These techniques have been explained in detail elsewhere [2]. In the current study, a para-sagittal approach was used and the needle was advanced in-plane with the ultrasound probe. The paravertebral space was easily identified and the injection of the local anesthetic was visualized in real-time and it was confirmed with pleural depression.

In a study by Cowie et al. [6], ultrasound-guided unilateral single-versus dual PVB injection techniques were compared by investigating the spread of the contrast dye in the paravertebral space. The single injection was applied at thoracic 6–7 segments with 20 mL contrast on one side, while the dual-injection was applied at thoracic 3–4 and 7–8 segments with 10 mL contrast for each. Contrast dye was demonstrated over three to four consecutive vertebral segments in 19 of 20 cadavers with no significant differences between the single- and dual-injection techniques. It was concluded that transverse in-plane ultrasound-guided needle insertion into the thoracic paravertebral space is both feasible and reliable, although the dual-injection technique at separate levels seems to cover more thoracic dermatomes with greater segmental intercostal spread compared to the single-injection technique. In the present study, the single unilateral injection technique at thoracic 7 vertebral level was used and there was seen to be a powerful analgesic effect after LC.

In the study by Naja et al., bilateral nerve stimulator-guided PVB with 0.3 mL/kg of a local anesthetic mixture containing lidocaine, bupivacaine, fentanyl and clonidine was applied at T5-6 level to patients undergoing LC. In the first 72 h postoperatively, improved pain relief was reported together with the use of less additional analgesia and a lower rate of complications such as nausea compared to the control group [8]. In a previous study assessing the radiographic and clinical distribution of single and multiple paravertebral injections using the same total volume of local anesthetic mixture, more reliable radiographic and clinical distribution was observed with the use of multiple paravertebral injections compared to the single-injection technique [16]. In the current study, unilateral PVB was applied at the T7 segment and significant improvements were determined, whether applied before or after surgery. No previous study could be found in literature which has compared the effectiveness of ultrasound-guided versus nerve stimulator-guided PVB during LC.

The main causes of pain after LC are thought to be pain arising from the incision site, pneumoperitoneum, and cholecystectomy. Factors such as diaphragmatic stretching, residual gas volume, type of gas, increased pressure due to pneumoperitoneum, the temperature of the insufflated gas, the length of the operation, and the rate of carbon dioxide insufflation might contribute to the pain after LC [2]. In the present study, after the preoperative application of unilateral PVB, none of the 30 patients in this group required any opioids during surgery. Furthermore, patients in both the preoperative and postoperative groups requested significantly less analgesia compared to the control group. Therefore, as even unilateral PVB can induce this analgesic effect, it can be considered that pneumoperitoneum, which is expected to induce diffuse pain bilaterally, does not significantly contribute to the pain during and after LC.

In another study by Naja et al., the authors compared the effectiveness of bilateral PVB applied preoperatively and postoperatively in 60 patients undergoing LC [7]. The PVB was performed under the guidance of a nerve stimulator at thoracic 5 and 6 level either prior to the induction of general anesthesia or immediately after the end of surgery. Patients treated with preoperative PVB were determined to have significantly lower VAS scores for pain at rest, on movement, and on coughing at 12 h postoperatively. Analgesia consumption was also lower in the preoperative PVB group. In the current study, the patients in the preoperative PVB group had significantly lower opioid requirements during surgery compared to the control and postoperative PVB groups, and significantly lower pain scores were detected in the preoperative PVB group compared to the postoperative PVB group. This difference might be partly explained by the local anesthetic used for PVB as bupivacaine alone was used in the present study, while a mixture of lidocaine, epinephrine, bupivacaine, fentanyl and clonidine was used in the previous study. It is not clear if this difference could be explained by the number of injection sites (unilateral versus bilateral) or the guidance used for PVB (ultrasound versus nerve stimulator).

Vascular puncture, hypotension, epidural or intratechal spread, pleural puncture and pneumothorax have been reported as possible complications during thoracic and lumbar PVB using a landmark technique facilitated by nerve stimulation. There has been reported to be an increased risk of vascular puncture and pneumothorax in bilateral compared to unilateral PVB [17]. Lonnqvist et al. [18] reported 10% block failure, 4.6% hypotension, 0.5% pneumothorax, and 3.8% vascular puncture in 362 patients [18]. The reported complication rates of unilateral and bilateral PVBs in 42 children and 620 adult patients by Naja et al. [17] are as follow: 6.1 and 0% block failure; 0.2 and 1.0% pneumothorax; 3.9 and 3.6% hypotension; 0.8 and 2.0% pleural puncture; 5.4 and 8.7% vascular puncture; and 1.9 and 3.1% hematoma, respectively. No urinary retention was reported. In the present study, using ultrasound guidance for thoracic unilateral PVB, no pleural puncture (verified with real-time ultrasound imaging) or subsequent pneumothorax, vascular puncture, or hypotension was observed. Furthermore, the incidence of postoperative nausea and vomiting was significantly lower in patients treated with PVB either pre- or postoperatively compared to patients who were administered general anesthesia only.

The current study had some limitations. The number of patients included in the study was relatively low compared to previous studies. Since we did not perform bilateral PVB, we were not able to compare unilateral PVB and bilateral PVB with regards to their effectiveness and complication rates. As the study included only patients who underwent elective cholecystectomy due to cholelithiasis, we were not able to generalize our results to all those undergoing cholecystectomy for any reason. Future studies with a larger number of patients are warranted, including patients with other biliary pathologies.

## 5. Conclusions

The present study revealed that ultrasound-guided PVB for LC is a useful and safe approach for pain management during and after LC for elective cholecystectomy due to cholelithiasis. PVB with a single unilateral injection can provide efficient analgesia. Furthermore, PVB combined with general anesthesia also reduced postoperative complications, and preoperative application of the block reduced the rate of intraoperative opioid requirement.

## Figures and Tables

**Table 1:** Clinical and demographic features of the groups.

Variables	Control	Preoperative Block	Postoperative Block	*p*-Value
**Age** (years)	43.83 ± 15.20	43.83 ± 12.86	49.70 ± 13.48	0.170 *
**Gender**				
Male	10 (33.3)	5 (16.7)	10 (33.3)	0.250 ^+^
Female	20 (66.7)	25 (83.3)	20 (66.7)	
**Height** (cm)	165 (155–182)	166 (155–184)	165 (150–184)	0.976 **
**Weight** (kg)	74.5 (60–87)	78.5 (62–93)	75 (62–93)	0.084 **
**ASA**				
1	9 (30)	15 (50)	13 (43.33)	0.610 ^+^
2	18 (60)	13 (43.33)	14 (46.67)	
3	3 (10)	2 (6.67)	3 (10)	
**Operation Duration** (min)	57.33 ± 10.23	58.33 ± 15.04	61.50 ± 15.20	0.528 *
**Intraoperative** **Opioid Requirement**				
Yes	30 (100)	0 (0)	30 (100)	**<0.001 ^++^**
No	0 (0)	30 (100)	0 (0)	
**Additional Analgesia**				
Yes	29 (96.67)	0 (0)	2 (6.67)	**<0.001 ^++^**
No	1 (3.33)	30 (100)	28 (93.33)	
**Nausea-Vomiting**				
Yes	14 (46.67)	6 (20)	5 (16.67)	**0.007 ^++^**
No	16 (53.33)	24 (80)	25 (83.33)	

Numerical variables were compared using One-Way Anova * or Kruskal Wallis ** tests after checking the normal distribution. Categorical variables were compared using the Chi-square test^+^ or Fisher’s Exact test ^++^. Data are given as mean ± standard deviation, median (25–75%) or number (*n*) and percentage (%).

## Abbreviations

LC	Laparoscopic cholecystectomy
PVB	Paravertebral block
US	ultrasound
